# Case series of acute appendicitis association with SARS-CoV-2 infection

**DOI:** 10.1186/s12879-021-05909-y

**Published:** 2021-02-25

**Authors:** Charlsea Prichard, Matthew Canning, Kindra McWilliam-Ross, John Birbari, William Parker, Lori Wasson, John W. Hollingsworth

**Affiliations:** 1 Texas Health Harris Methodist Hospital Fort Worth, Fort Worth, USA; 2 Texas Christian University School of Medicine and Texas Pulmonary & Critical Care Consultants PA, 1201 Fairmount Ave, Fort Worth, TX 76104 USA; 3 University of Tulsa, Tulsa, USA

**Keywords:** COVID19, Appendicitis, SARS-CoV-2

## Abstract

**Background:**

Describe the indications for surgical interventions in asymptomatic patients with SARS-CoV-2. We are unaware of previous reports of an association between SARS-CoV-2 and acute appendicitis.

**Methods:**

We performed a single institution retrospective review of SARS-CoV-2 pre-procedure testing and indications for surgical intervention. Statistical comparisons were performed using Chi Square analysis or two-tailed Student T test.

**Results:**

We report a high prevalence of SARS-CoV-2 in both all testing and pre-procedure testing during the enrollment period. We observe a high prevalence of acute appendicitis among patients identified to be SARS-CoV-2 positive during pre-procedure testing and without recognized symptoms of COVID19.

**Conclusion:**

We report a previously unrecognized association between SARS-CoV-2 and acute appendicitis.

## Background

Recent observations with abdominal imaging in SARS-CoV-2 (severe acute respiratory syndrome coronavirus 2) infection demonstrate that bowel abnormalities are common [1]**.** Previous studies suggest a relationship between upper respiratory viral diseases and appendicitis [2]. A recent case report describes patients presenting with appendicitis-like symptoms, but ultimately was discovered to be SARS-CoV-2 positive with coronavirus disease 2019 (COVID19) and not have appendicitis [3]. SARS-Cov-2 pandemic has been reported to impact management of acute appendicitis with possibly fewer cases presenting to the hospital [4], a delay the time of diagnosis, increased frequently peritonitis [5] and more severe septic abdominal diseases [6]. These previous reports suggest a potentially harmful impact of SARS-CoV-2 pandemic on access to emergency surgery services. However, to our knowledge, an association between testing positive for SARS-CoV-2 and presentation to the hospital with acute appendicitis has not previously been reported.

It is recognized that patients with asymptomatic SARS-CoV-2 infection undergoing elective surgeries experience both respiratory-related complications and high mortality [7]. For this reason, our hospital has adopted a policy to test patients undergoing non-emergent interventional procedures for SARS-COV-2. We report our observations supporting an association between acute appendicitis and SARS-CoV-2.

## Methods

To determine whether patients with appendicitis were over-represented in patients unknown to have COVID-19 but testing positive for SARS-CoV-2, we collected data on SARS-CoV-2 testing done between May 1, 2020 and July 22, 2020 to be included in this this retrospective cross-sectional observational study. We report results from testing performed at a single center, Texas Health Harris Methodist Hospital Fort Worth. No patients were excluded from the analysis. For this study, we reviewed the individual records of patients undergoing pre-procedure testing for SARS-CoV-2. Patients in this cohort did not present with a clinical suspicion of COVID, but rather were being evaluated for interventional procedures in an effort determination of peri-procedural risk. Clinical decision for operative intervention was not based on SARS-CoV-2 results. Pathology was reviewed by two pathologists. Statistical comparisons were performed using Chi Square analysis or two-tailed Student T test. We received IRB approval for retrospective chart review. The study was approved by the University of Texas Southwestern Medical Center Institutional Review Board (STU-2020-0405).

## Results

**Fig. 1:**
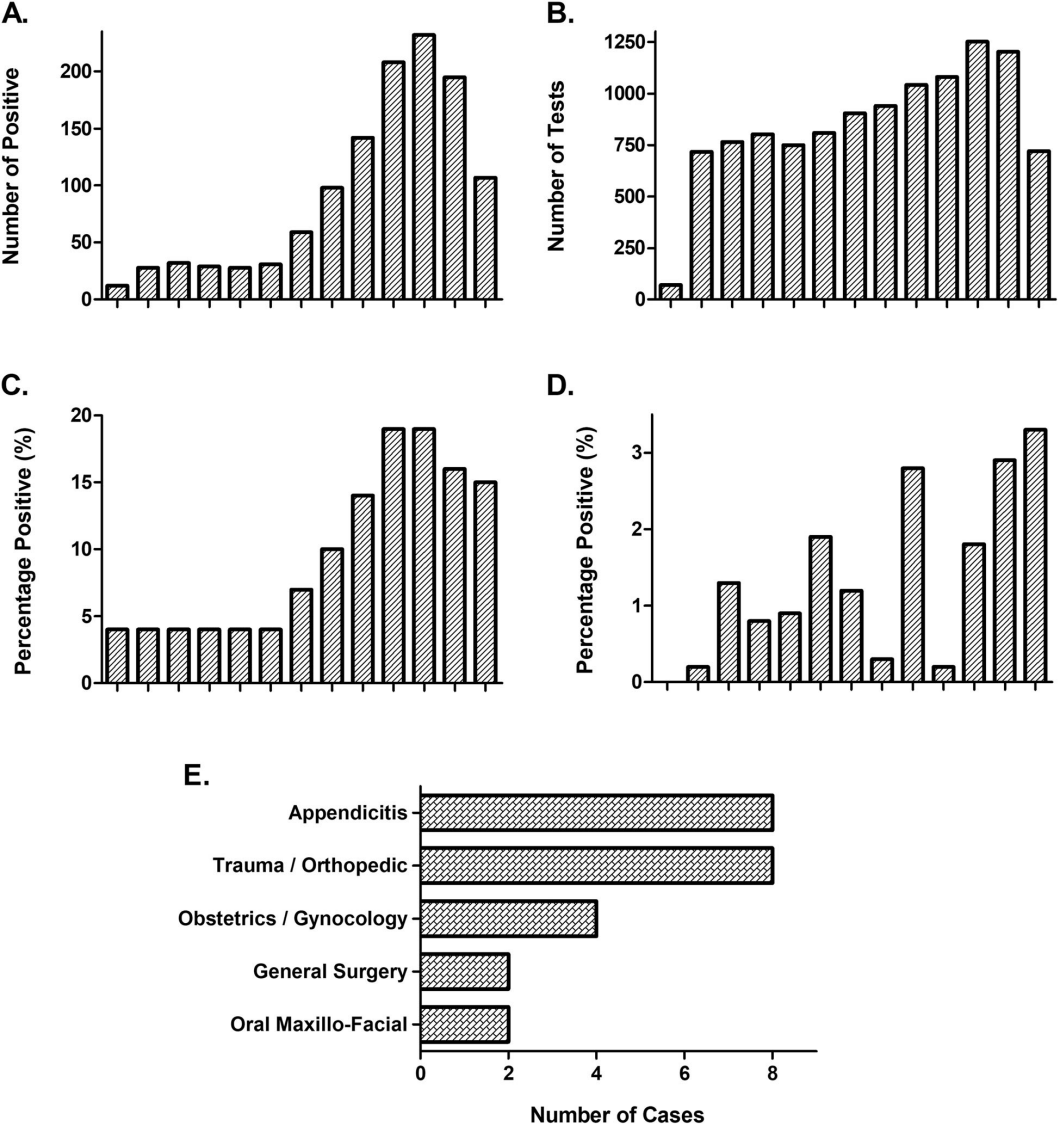
Demographics of COVID19 testing and diagnosis in asymptomatic SARS-CoV-2 positive cases requiring surgery. **a** The number of total PCR positive cases over the enrollment period. **b** The number of tests performed during each time period. **c** The overall rate of detecting positive PCR test for COVID19 over time. **d** The rate of detecting positive PCR test for COVID19 in pre-procedure testing over time. **e** We report a total of 24 patients requiring emergent surgery that were SARS-CoV-2 positive on pre-procedure testing. Appendicitis represented 33.3% (8/24) of patients that were SARS-CoV-2 positive and required emergent surgery

There was a total of 11,058 tests performed with 6047 pre-procedure tests performed on patients presenting for interventional procedures. A total of 1.4% (87/6047) pre-procedure patients tested positive for SARS-CoV-2 during the period of enrollment. Our hospital has observed a surge in number of COVID19 cases during June–July 2020 (Fig. 1 a). While we have slightly increased our capacity of testing during this time period (Fig. 1 b), we have an overall 9.4% positive rate with a dramatic increase in recent weeks (0–19% daily-positive rate) (Fig. 1 c). These findings are consistent with a surge in cases in our region of the country. Our hospital has adopted a protocol to test all patients prior to invasive procedures. This strategy provides some insight into the community prevalence of asymptomatic disease as these patients did not present with typical symptoms of COVID19. ln recent weeks, we observed an increase in percentage of positive SARS-CoV-2 tests prior to procedures. Most recent observations demonstrate a rate of 3.3% SARS-CoV-2 positive in patients presenting for procedures without previously recognized symptoms of infection (Fig. 1 d).

Previous reports suggest potentially harmful impact of SARS-CoV-2 pandemic on emergency surgery services. During the enrollment period during the pandemic, our hospital observed an increased number of acute appendicitis, when compared to the prior 3 years. Appendectomies represent 3.1% (83 / 2646) of cases during the enrollment period in 2020, when compared to an average of 2.4% (72 / 3002) during the same period of time in 2017–2019. Furthermore, the diagnosis of appendicitis appeared over-represented when reviewing all cases of SARS-CoV-2 positive from pre-procedure testing when compared to other diseases. We identified through pre-procedure SARS-CoV-2 testing that a diagnosis of acute appendicitis is much more likely to be positive for SARS-CoV-2, when compared to all other patients presenting for interventional procedures (10.8% vs 1.3%; *p* < 0.001). A total of 9.2% (8/87) of patients testing positive for SARS-CoV-2 had acute appendicitis. While 27.6% (24/87) of patients whom tested positive for SARS-CoV-2 required emergent surgery, a remarkable 33.3% (8/24) of patients, whom both tested positive for SARS-CoV-2 and required surgery, had acute appendicitis (Fig. 1 e**).** There was a total of 83 appendicitis cases at our hospital during the enrollment period and 10.8% (9/83) were COVID19 positive, which is much higher than the overall 1.4% prevalence of disease observed in overall pre-procedure testing.

When comparing patients with appendicitis and positive for SARS-CoV-2 (*N* = 8) to other patients requiring surgery and positive for SARS-CoV-2 (*N* = 16), we observed lower percentage of lymphocytes (10.9% versus 18.6%, *p* < 0.05). Pathologist confirmed diagnosis of suppurative appendicitis with pathological review of 88.9% (8/9) cases with one case was treated medically. We observe perforation in 38% (3/8) of patients with appendicitis with SARS-CoV-2 versus 25% (2/8) in control appendicitis cases during the same time period who tested negative for SARS-CoV-2. We did not observe differences in lymphoid aggregates and there was no evidence of viral inclusions.

## Discussion

Together our initial observations support a surge of COVID19 cases in North Texas, USA with an increased percentage of overall positive tests and an increase in positive tests in patients scheduled for procedures. We are unaware of redistribution of patients between hospitals in our region of the country resultant from the pandemic. We observed a slight increased number of acute appendicitis cased when compared to the previous 3 years. While there were no observed differences in pathology, our findings support an unanticipated association between SARS-CoV-2 and acute appendicitis.

## Conclusion

Patients with acute appendicitis who undergo pre-procedure testing have a significant increased likelihood of having unrecognized COVID19. These findings suggest that SARS-CoV-2 infection may contribute to the pathogenesis of acute appendicitis. We recommend testing patients whom present with acute appendicitis for SARS-CoV-2. Additional studies may be justified to determine whether SARS-CoV-2 contribute to the pathogenesis of acute appendicitis.

## Data Availability

The datasets used and analysed during the current study are available from the corresponding author on reasonable request.

## Abbreviations

COVID19: Coronavirus disease 2019
SARS-CoV-2: Severe acute respiratory syndrome coronavirus 2
